# How Doctors View and Use Social Media: A National Survey

**DOI:** 10.2196/jmir.3589

**Published:** 2014-12-02

**Authors:** James Brown, Christopher Ryan, Anthony Harris

**Affiliations:** ^1^Western Clinical SchoolDepartment of PsychiatryUniversity of SydneyWestmeadAustralia

**Keywords:** social media, Internet, professional practice, health communication, ethics, health policy, patient-physician relations

## Abstract

**Background:**

Doctors are uncertain of their ethical and legal obligations when communicating with patients online. Professional guidelines for patient-doctor interaction online have been written with limited quantitative data about doctors’ current usage and attitudes toward the medium. Further research into these trends will help to inform more focused policy and guidelines for doctors communicating with patients online.

**Objective:**

The intent of the study was to provide the first national profile of Australian doctors’ attitudes toward and use of online social media.

**Methods:**

The study involved a quantitative, cross-sectional online survey of Australian doctors using a random sample from a large representative database.

**Results:**

Of the 1500 doctors approached, 187 participated (12.47%). Most participants used social media privately, with only one-quarter not using any social media websites at all (48/187, 25.7%). One in five participants (30/155, 19.4%) had received a “friend request” from a patient. There was limited use of online communication in clinical practice: only 30.5% (57/187) had communicated with a patient through email and fewer than half (89/185, 48.1%) could offer their patients electronic forms of information if that were the patients’ preference. Three in five participants (110/181, 60.8%) reported not being uncomfortable about interacting with patients who had accessed personal information about them online, prior to the consultation. Most of the participants (119/181, 65.8%) were hesitant to immerse themselves more fully in social media and online communication due to worries about public access and legal concerns.

**Conclusions:**

Doctors have different practices and views regarding whether or how to communicate appropriately with patients on the Internet, despite online and social media becoming an increasingly common feature of clinical practice. Additional training would assist doctors in protecting their personal information online, integrating online communication in patient care, and guidance on the best approach in ethically difficult online situations.

## Introduction

There is widespread international disquiet about the ramifications of online and social media on clinical practice [1-7]. Doctors are uncertain of patient expectations [8,9], and of their ethical and legal obligations when using online communication [10,11]. However, there has been limited quantitative research into doctors’ usage of this technology or their attitudes toward it [12-14]. As a consequence, professional guidelines aimed at providing ethical and practical oversight have been written with little empirically derived data.

The current standards of professional communication in medicine were developed with traditional face-to-face consultations as the template. This model of professionalism has not transferred easily to an online environment and doctors are being left to act using their own intuition as new online ethical dilemmas arise [15-17]. For example, there is uncertainty as to how a doctor ought to respond to a “friend request” from a patient via social media, despite poor handling of the situation potentially affecting the viability of the therapeutic relationship [18]. Intuitions may differ between different age cohorts. Younger doctors, who use social media frequently [19], may be more comfortable communicating online [20,21] than senior doctors, who have limited familiarity with the technology.

While concerns about online searching [22,23], privacy [24], and professionalism [25] have been suggested as barriers to the use of social media by doctors, the slow adoption of social media also represents a possible lost opportunity for the medical profession at large to engage with patients and the general community. There is a need to examine the impact of the use of social media on physicians’ knowledge, attitudes, skills, and behaviors in practice [26] and the influence of concerns about ethics, professionalism, and privacy upon that use [25,27].

The aim of our study was to investigate and quantify the current use of social and online media by doctors and gauge attitudes toward possible professional dilemmas when communicating with patients online in order to assist in the improvement of social media guidelines for effective use in clinical practice.

## Methods

### Participants

We selected a random sample of 1500 medical practitioners, who had email addresses on the Australasian Medical Publishing Company (AMPCo) database. The AMPCo database holds the contact details of 65,536 doctors, representing 93% of all doctors in Australia [28]. A total of 49% of the database had email addresses. AMPCo extracted all names using an automated randomization process that selects contacts proportionally from their database by age, gender, and location. Study investigators were unaware of the identity of any participant. This sample size was selected following review of expected response rates to unsolicited, non-incentivized, online medical research surveys [27,29-32] and in consultation with other large Australian-based surveys.

All participants were sent an invitation email including a link to an online survey and a participant information sheet explaining the rationale for the study. A repeat email was sent to non-responders 4 weeks later. No financial or educational incentives were provided to participants. Data was collected for 8 weeks from October to December 2013. All responses were automatically recorded via the survey platform and downloaded into an SPSS database. The study was approved by the University of Sydney Human Research Ethics Committee.

### Questionnaire

The survey questionnaire was developed after a literature review following the Checklist for Reporting Results of Internet E-Surveys (CHERRIES) guideline [33]. It covered five broad areas of patient-doctor online interaction: (1) current participant usage, (2) general online behavior, (3) doctors’ personal information online, (4) patients’ information online, and (5) appropriate patient-doctor online interaction. Questions were multiple choice. A draft questionnaire was piloted and revised to a final survey of 36 items (plus demographic information) that took approximately 10 minutes to complete (see Multimedia Appendix 1).

### Analysis

Data were analyzed with IBM SPSS Statistics 20 statistical software. Statistical approach was based upon distribution of responses. We compared proportions using chi-square analysis; means were compared using *t* tests. Logistic regression was used to estimate the association between social media use and doctor profile.

## Results

### Participants

Of the 1500 doctors who received the invitation email, 190 logged on to the survey and 187 completed it yielding a response rate of 12.47%. The participants were drawn from all states and territories of Australia and were representative of a broad range of specialties. The demographics of the participants were largely similar to that of the AMPCo database (Table 1), although a larger than expected proportion of participants (33.1% vs 23% [34]) identified as working in a rural location. The survey responses were generally consistent across all demographic groups. We have noted the exceptions to this in the text below.

**Table 1:** Participant demographics (n=187).

Demographics		n (%)
**Current age (in years)**		
	Less than 25	2 (1.1)
	25-35	43 (23.0)
	36-45	40 (21.4)
	46-55	45 (24.1)
	56-65	29 (15.5)
	66-75	11 (5.9)
	Greater than 75	5 (2.7)
	Missing	12 (6.4)
**Gender**		
	Male	95 (50.8)
	Female	80 (42.8)
	Missing	12 (6.4)
**Rural or metropolitan**		
	Rural	58 (31.0)
	Metropolitan	117 (62.6)
	Missing	12 (6.4)
**Professional role**		
	Intern / junior medical officer	17 (9.0)
	Doctors undertaking specialty training	36 (19.2)
	Specialist physicians	72 (38.5)
	General practitioner	46 (24.6)
	Not presently practicing	1 (0.6)
	Retired	2 (1.1)
	Other	1 (0.6)
	Missing	12 (6.4)
**Private or public practice**		
	Private only	65 (34.8)
	Public only	71 (38.0)
	Both public and private	37 (19.8)
	Not applicable	2 (1.1)
	Missing	12 (6.4)
**Years since graduation**		
	Less than 10	48 (25.7)
	10-19	39 (20.9)
	20-29	37 (19.8)
	30-39	37 (19.8)
	40-49	9 (4.8)
	Greater than 50	5 (2.7)
	Missing	12 (6.4)

### Current Online and Social Media Use by Doctors

Most participants used social media at least once a week and only one-quarter did not use any social media websites at all (48/187, 25.7%). The most commonly used platform was Facebook (112/187, 59.9%). Most participants (147/187, 78.6 %) used social media in non-work hours, and of those, 16.3% (24/147) used social media more than 1 hour per day. In comparison, 38.0% (71/187) of participants used social media in work hours, with only 4.2% of those using the technology more than 1 hour per day.

There was a linear relationship between increasing age and decreasing social media use (OR 10.3, 95% CI 2.8-42.4). All interns, junior medical officers, and doctors undertaking specialty training used some form of social media compared with 72.2% (52/72) of specialist physicians and 69.6% (32/46) of general practitioners (*P*<.001).

### Attitudes Toward, and Utilization of, Online Patient-Doctor Communication

Attitudes toward the use of social media with patients were divided. Although 67.0% (124/185) of participants agreed it might be appropriate for a doctor to interact with their patient via email, only 30.5% (57/187) of participants volunteered that they had done so. Only 1 of the 187 respondents had used social media (eg, Twitter or Facebook) to communicate with patients and only 21.2% (38/179) believed it would be appropriate to do so. Over one-third (63/185, 34.1%) of participants did not have a website or online presence for their practice, and over half (96/185, 51.9%) could not offer their patients electronic forms of information if that was a patient’s preference.

In contrast to these low rates of online communication, doctors frequently spoke to their patients about online resources; 69.7% (129/185) had discussed online information sources (such as websites about their disease), though fewer had discussed social media resources such as online support groups (73/185, 39.5%). General practitioners were far more likely than specialist physicians to speak with their patients about Internet usage and online resources (44/46, 95.7% vs 43/72, 59.7%, *P*=.005). Rural and regional doctors were also more likely to have discussed Internet resources with their patients than their urban colleagues (45/58, 77.6% vs 77/117, 65.8%, *P*=.006).

### Patient-Doctor Interaction on Facebook

Despite the very low rates of social media use in a professional setting, there were high rates of use in private with women being much more likely to use Facebook than men (59/80, 73.8% vs 49/95, 51.6%, *P*=.03). One in five participants (30/155, 19.4%) had received a friend request from a patient they only knew and interacted with professionally. When asked how they would respond to a patient who had sent them a friend request, participants were split in their responses (Table 2). Most commonly they would decline the request and do nothing more (54/155, 34.8%). Only 2.6% (4/155) would accept the friend request. Over half (89/155, 57.4%) thought it appropriate for a doctor to maintain a personal Facebook profile, though less than one-quarter (37/155, 23.9%) were comfortable with a patient being able to access content about the doctor posted on that page (such as photos posted by others).

**Table 2:** Doctors’ response to friend request from a patient (n=155).

How would you respond to a patient who sent you a friend request on Facebook?	n (%)
Accept the request	4 (2.6)
Decline the request and do nothing more	54 (34.8)
Decline the request and send a private message explaining why	18 (11.6)
Decline the request and discuss at the next consultation	44 (28.4)
Do nothing	35 (22.6)

### Protection of Personal Information Online

Most participants (110/181, 60.8%) reported they would not be comfortable interacting with a patient who had accessed personal information about them online prior to the consultation and 17.1% (31/181) of participants had experienced someone else posting information online about them, which they would not want patients to see. Although not common, some participants had interacted with patients who described information about them which they had not made available and which the patient found online (15/181, 8.3%) or on social media (3/181, 1.7%).

Few doctors were able to adequately protect their information online. While most participants were aware of the results that appear when they searched on the Internet for their full name (117/181, 64.6%), and the majority (107/155, 69.0%) have adjusted privacy settings to limit access to their information, a much smaller proportion took measures to control their online profile (65/181, 35.9%). The older participants were, the less likely they were to know how to remove photos of themselves they wouldn’t want patients to see. No participant aged over 65 years knew how to do this, compared with 7% (3/45) aged 46-55 years, and 50% (1/2) aged under 25 years (*P*=.01). Females were more likely than males to control and curate their online profile, including adjusting privacy settings (38/80, 48% vs 25/95, 26%, *P*=.05).

Most participants (119/181, 65.8%) were hesitant to immerse themselves more fully in social media and online communication due to worries about public access and legal concerns.

### Other Ethical Dilemmas

Doctors were also unsure if they had a duty to rebut inappropriate or inaccurate information posted online; with 38.1% (69/181) saying that doctors did and 29.3% (53/181) saying they did not, with the rest being undecided. There was no consensus about the appropriateness of accessing publically available information about a patient and whether to broach that with the patient (Table 3).

Even in an emergency, 26.7% (48/180) of participants would not use publicly available online information (eg, a patient’s Facebook page for information regarding a suicide attempt). Despite the split responses, 16.1% (29/180) of participants had already searched for information about a patient online (Table 4).

**Table 3:** Doctors’ use of publically available patient information (n=180).

If you were to use publicly available online information about a patient to assist in their treatment, would you, as the doctor, discuss it with the patient?	n (%)
Yes, always	72 (40.0)
Yes, sometimes	18 (8.9)
Yes, rarely	4 (2.2)
No	4 (2.2)
Unsure	37 (20.6)
I would not use publicly available online information	47 (26.1)

**Table 4:** Examples of online ethical dilemmas.

Dilemma	Yes	No	Unsure
	n (%)	n (%)	n (%)
Do doctors have a duty to rebut inappropriate or inaccurate health information posted online? eg, a blogger saying that sex without a condom is safe.	69 (38.1)	53 (29.3)	59 (32.6)
Have you at any time searched for publicly available online information about a patient? eg, “Googled” a patient to find more information about them.	29 (16.1)	150 (83.3)	1 (0.6)
Is it appropriate for doctors to look up publicly available online information about a patient in an emergency? eg, searching a patient’s Facebook page for information following a suicide attempt.	68 (37.8)	48 (26.7)	64 (35.6)
Is it appropriate for doctors to look up publicly available online information about a patient as part of regular clinical practice? eg, monitoring a pro-anorexia forum for posts made by one of your patients.	30 (16.7)	77 (42.8)	73 (40.6)

## Discussion

### Current Usage of Online and Social Media by Doctors is Limited

Our results confirm our hypothesis that Australian doctors have yet to fully integrate online communication and social media into their clinical practice, and many are unable to protect their personal information online.

Although Australian doctors frequently used social media in their own private lives, their use during their working day is minimal. This reflects similar usage patterns noted among US doctors [27]. Few changes have been made to integrate online communication into clinical practice (including resources as basic as email), despite this being a common expectation for client communication in most other professions. Over half of participants are unable to send information electronically; many practices cannot offer their patients the option of electronic communication at all, even if that is their preference. As a result, patients are not even given the choice of online communication in most medical facilities.

The relationship between age and social media use is not surprising. Younger doctors have grown up with online communication, and frequent personal use may have instilled confidence in their ability to navigate any potentially hazardous ethical dilemmas. In comparison, older doctors have not been as involved in the progressive integration of social media into daily life, nor the increasing volume of its use. As a result, social media fluency can vary greatly within a cohort of doctors within the same practice or hospital, interacting with the same patients.

In contrast to this low professional usage, many doctors are discussing Internet and social media resources with their patients. General practitioners (44/46, 96%) and rural doctors (44/58, 76%) report an extremely high rate of discussion about online resources, perhaps reflecting the central role of online health resources as part of primary care, especially in geographically distant centers, and the potential for further integrating online and social media in other areas of medical practice.

### Doctors Are Unsure as to How to Respond to Online Ethical Dilemmas

Although patient-doctor online communication is currently limited, doctors are still encountering online ethical dilemmas. One in five doctors had already found themselves in situations where the traditional boundaries of the doctor-patient relationship had been stretched by a friend request on social media. This figure was considerably lower than the 34.5% of practicing physicians who reported receiving such a request from a patient reported in a recent US study [27]. When questioned about how they would react in such a situation, Australian doctors were evenly distributed as to whether they would respond to or ignore the request and how they would do that, reflecting the absence of consensus on how best to approach such a situation.

Equally problematic was the question of whether it is appropriate to view publically available online information about patients, even in an emergency, and whether patients should be made aware that this information had been accessed. It is interesting to reflect upon whether a post on Facebook about a suicide attempt is any different to a written suicide note, as few would have any ethical concerns about reading the latter. Perhaps most surprisingly, given the readiness to discuss online resources with patients, doctors were unwilling to become involved in online discussions about the accuracy or appropriateness of online material or blogs. This may in turn be related to their uncertainty about the Internet and social media.

### Doctors’ Personal Information Is Not Adequately Protected

Doctors were concerned about legal issues when communicating with patients online and reported that privacy and legal concerns were driving their reluctance to participate more fully in social media. These concerns are comparable to those shown in other countries [27] and remain unaltered despite the development of guidelines for social media published by professional organizations before and during the study period [35-39]. The majority of doctors (125/181, 69.0%) stated they would be uncomfortable interacting with patients who had accessed online information about them prior to a consultation. Yet few take specific measures to manage and edit the information available about themselves online. Only one in five (39/181, 21.6%) know what to do if a compromising photo of them is posted without their permission, with 17.1% saying that information about them they wouldn’t want patients to see had already been posted. This limited competency in managing the online information available about them means that the possible advantages of an Internet presence are lost and increases the risk of negative experiences, further reinforcing avoidance of the use of social media.

Many doctors believe they should not have a personal profile, to avoid such dilemmas coming about. This only further marginalizes doctors from developing experience and fluency in the use of the technology and ignores the fact that as time goes on a doctor’s online profile will be developed even without their participation via the increasing use of third-party review sites [40,41] such as RateMDs.com and Google Reviews.

What is most evident from the results of this study is that the permeation of online and social media into everyday life is placing doctors in new situations that they find difficult to navigate. It is clear that the standards and practices that have previously guided everyday patient-doctor interactions are being placed in a new light as the profession adapts old understandings to new communication tools. For some, social media may simply be another innocuous form of communication, like a phone call or a text message. While others may consider the personal information about themselves available on social media to be only appropriate for their closest friends and family.

### Limitations of the Research

The findings of the study are weakened by the modest response rate of 12.5%, though this is similar to that obtained by other studies using online collection [27,42]. As we only invited doctors via email this will have removed a number of participants who do not have an email address (51% of the AMPCo database do not have recorded email addresses). It is also likely that we elicited responses from doctors who are more interested in social media, potentially biasing our sample. This is likely to be in the direction of a group more cognizant with the use of the Internet and social media. Nevertheless, because our findings demonstrate a range of attitudes toward appropriate social media use with a population more engaged with social media, our study, if anything, likely underestimates doctors’ discomfort with using social media.

### Future Directions

This study reveals two key areas where research must be directed. First, consensus must be reached on how doctors should behave online. This requires an expanded investigation of global and country-specific standards for online communication and for professional bodies to make these expectations included as part of training and professional development. Second, current ethical and professional guidelines may be ineffective in guiding appropriate patient-doctor interaction online and informing doctors in how to protect their personal information. Therefore, a reappraisal of how these guidelines are promulgated needs to occur. Change in behavior in such a central part of medicine will require active engagement rather than the passive diffusion of guidelines. The researchers intend to repeat the study in 5 years and provide the survey tool to assist further social media research internationally.

### Conclusion

Our study is a comprehensive description of current online and social media behavior of doctors. It underlines the continuing need to improve the online capabilities of doctors and refine online and social media guidelines for doctors, which have so far done little to improve the uncertainty of doctors online. It finds a surprising reluctance to engage with the new media despite the demands of the community. The results will also allow us to map emerging trends in social and electronic media use, bringing future ethical issues to light as online communication becomes more and more relevant to clinical practice.

## Multimedia Appendix 1

Survey tool used in the research.

## Abbreviations

AMPCo: Australasian Medical Publishing Company
