# Supplementary material for: How Doctors View and Use Social Media: A National Survey
Source: J Med Internet Res. 2014 Dec 2;16(12):e267. doi: 10.2196/jmir.3589 (PMC4275505; doi:10.2196/jmir.3589)
Supplement: Supplementary file 1 [file jmir_v16i12e267_app1.pdf]

<b>Australian doctors' attitudes towards, and usage of, online and social

1. Your current online usage

Thank you for taking the time to fill out this survey. The following questions pertain to your usual pattern of internet behaviour and usage. When responding, please imagine your behaviour and usage in a typical week.

The survey will take less than 10 minutes to complete.

1. Which of the following social media do you use at least once a week (check all that apply):

- [ ] Facebook
- [ ] Twitter
- [ ] YouTube
- [ ] Google+ (not the search engine itself)
- [ ] Blogging service (for example Wordpress, Tumblr, etc.)
- [ ] LinkedIn
- [ ] MySpace
- [ ] ResearchGate or Academia
- [ ] **I do not use any of these websites**

Other (please specify)

2. How many *work* hours do you use the above social media per day?

- ( ) Never
- ( ) 0-1 Hours
- ( ) 1-3 Hours
- ( ) 3-6 Hours
- ( ) >6 Hours

3. How many *non-work* hours do you use the above social media per day?

- ( ) Never
- ( ) 0-1 Hours
- ( ) 1-3 Hours
- ( ) 3-6 Hours
- ( ) >6 Hours

2. General online behaviour

1. In your role as a doctor, do you interact with patients via email or through social media?

- ( ) Yes
- ( ) No

2. General online behaviour

1. Through which of the following do you interact with patients? (check all that apply)

- [ ] Email
- [ ] Facebook
- [ ] Twitter

Other (please specify)

2. Which of the following online media does your private practice or other place of work currently have for patients to access? (check all that apply)

- [ ] A website
- [ ] Blog
- [ ] A Facebook page
- [ ] *My practice does not have a website or other electronic presence*
- [ ] Other (please specify)

3. Have you been questioned by patients about online means of contacting you (beyond a professional email address)?

○ Yes ○ No ○ Unsure

4. Do you ever discuss internet usage with your patients? e.g. how to access online information about their disease.

○ Yes ○ No ○ Unsure

5. If a patient describes a preference for receiving information electronically only, can you do this?

○ Yes ○ No ○ Unsure

6. Is it ever appropriate for a doctor to interact with his or her patients professionally through email?

○ Yes ○ No ○ Unsure

7. Do you ever discuss social media usage with your patients? e.g. online support groups for people with haemochromatosis.

○ Yes ○ No ○ Unsure

8. Do you share other people's online content through social media? e.g. online posts made by other doctors.

○ Yes ○ No ○ Unsure

3. Your personal information online

1. Are you aware of the results that appear when you Google your full name?

○ Yes ○ No ○ Unsure

2. Do you currently take measures to curate and control your online profile?

○ Yes ○ No ○ Unsure

3. Do doctors have a duty to rebut inappropriate or inaccurate health information posted online? e.g. a blogger saying that sex without a condom is safe.

○ Yes ○ No ○ Unsure

4. Has information about you been put online by others that you would not want patients to see? e.g. photos and/or videos.

○ Yes ○ No ○ Unsure

5. If there were photos of you online that you didn't want patients to see, would you know what to do in order to remove them?

○ Yes ○ No ○ Unsure

6. At any point, has a patient described information that they have found out about you online, which you have not knowingly authorised to be publicly available? e.g. information posted on a website found through Google.

○ Yes ○ No ○ Unsure

7. At any point, has a patient described information that they have found out about you on *social media*, which you have not knowingly authorised to be publicly available? e.g. information posted on your Facebook profile.

○ Yes ○ No ○ Unsure

8. Have you at any time had to remove online content due to it being potentially seen by patients?

○ Yes ○ No ○ Unsure

9. Would you be comfortable interacting with a patient who has accessed personal information about you online, prior to a consultation?

○ Yes ○ No ○ Unsure

10. Have concerns about public access made you hesitant to immerse yourself more fully into social media and online communication opportunities?

○ Yes ○ No ○ Unsure

4. Your patients' information online

1. Are you concerned of possible legal issues around interacting with patients online?

○ Yes ○ No ○ Unsure

2. Have you at any time searched for publicly available online information about a patient? e.g. 'Googled' a patient to find more information about them.

- Yes
- No
- Unsure

3. Is it appropriate for doctors to look up publicly available online information about a patient in an emergency? e.g. searching a patient's Facebook page for information following a suicide attempt.

- Yes
- No
- Unsure

4. Is it appropriate for doctors to look up publicly available online information about a patient as part of regular clinical practice? e.g. monitoring a pro-anorexia forum for posts made by one of your patients.

- Yes
- No
- Unsure

5. If you were to use publicly available online information about a patient to assist in their treatment, would you, as the doctor, discuss it with the patient?

- Yes, always
- Yes, sometimes
- Yes, rarely
- No
- Unsure
- I would not use publicly available online information

5. Appropriate patient-doctor online interaction

1. With adequate training, would you be comfortable conducting a consultation with a patient via Skype (or other online telecommunications)?

- Yes
- No
- Unsure

2. With adequate training, would you be comfortable presenting online video content, about yourself and services, for patients?

- Yes
- No
- Unsure

3. Is it ever appropriate for a doctor to interact with his or her patients professionally through social media?

- Yes
- No
- Unsure

4. The following questions regard the use of Facebook. Are you familiar with Facebook?

- Yes, I am familiar with Facebook
- No, I am not familiar with Facebook

5. Appropriate patient-doctor online interaction

1. Is it appropriate for a doctor to maintain a personal Facebook profile that could inadvertently be found by patients?

- Yes
- No
- Unsure

2. Would you be comfortable with a patient being able to access content of you posted on your own page? e.g. Facebook photos posted by others that you are identified in.

- Yes
- No
- Unsure

3. Have you at any point received a ‘friend request’ on Facebook from someone you only know and interact with as their doctor?

- Yes
- No
- Unsure

4. Have you adjusted the privacy settings in Facebook to limit public access to you information?

- Yes
- No
- Unsure

5. How would you respond to a patient who sent you a friend request on Facebook? Select from below:

- Accept the request
- Decline the request and do nothing more
- Decline the request and send a private message explaining why
- Decline the request and discuss at the next consultation
- Do nothing

Other (please specify)

6. Demographics

1. Current age (in years):

- <25
- 25-35
- 36-45
- 46-55
- 56-65
- 66-75
- >75

2. Gender:

- Male
- Female

3. State:

- ACT
- NSW
- QLD
- SA
- TAS
- VIC
- WA
- NT

Other (please specify)

4. Are you based in a rural/regional location?

- Yes
- No

5. Position:

- Intern
- Fellow
- GP
- RMO
- Career medical officer
- Not presently practising
- Registrar
- Consultant
- Retired

Specialty (if applicable):

6. Private or public practice:

- Private
- Public
- Both
- N/A

7. Years since graduation:

- <10
- 10-19
- 20-29
- 30-39
- 40-49
- >50
